# The efficacy of intrathecal methyl-prednisolone for acute spinal cord injury: A pilot study

**DOI:** 10.1016/j.heliyon.2023.e15548

**Published:** 2023-04-17

**Authors:** Ali Meshkini, Mohammad Kazem Sarpoolaki, Ali Vafaei, Farhad Mirzaei, Abolfazl Badripour, Ebrahim Rafiei, Morteza Khalilzadeh, Mohammad Reza Fattahi, Arad Iranmehr

**Affiliations:** a Neurosurgery Department, Tarbiz University of Medical Sciences, Iran; b Neurological Surgery Department, Imam Khomeini Hospital Complex (IKHC), Tehran University of Medical Sciences, Iran; c Experimental Medicine Research Center, Department of Pharmacology, Tehran University of Medical Sciences, Iran; d Brain and Spinal Cord Injuries Research Center, Neuroscience Institute, Tehran University of Medical Sciences, Iran; e Tehran University of Medical Sciences (TUMS), Iran; f Sina hospital, Hasanabad, Tehran, Iran

**Keywords:** Methyl-prednisolone, Spinal cord injury, ASIA score, Frankel score, Oxidative stress

## Abstract

**Study design:**

Randomized clinical trial.

**Objectives:**

To evaluate the safety and effectiveness of intrathecal methyl-prednisolone compared to intravenous methyl-prednisolone in acute spinal cord injuries.

**Setting:**

Imam Reza Hospital, Tabriz University of Medical Sciences.

**Methods:**

Patients meeting our inclusion and exclusion criteria were enrolled in the study and divided randomly into two treatment arms: intrathecal and intravenous. Standard spinal cord injury care (including surgery) was given to each patient based on our institutional policy. Patients were then assessed for neurological status (based on ASIA scores, Frankel scores) and complications for six months and compared to baseline status after injury. To better understand the biological bases of methyl-prednisolone on spinal cord injuries, we measured two biomarkers for oxidative stress (serum malondialdehyde and total antioxidant capacity) in these patients at arrival and day three after injury.

**Results:**

The present study showed no significant difference between the treatment arms in neurological status (sensory scores or motor scores) or complications. However, the within-group analysis showed improvement in neurological status in each treatment arm within six months. Serum malondialdehyde and total antioxidant capacity were analyzed, and no significant difference between the groups was seen.

**Conclusion:**

This is the first known clinical trial investigating the effect of intrathecal MP in acute SCI patients. Our finding did not show any significant differences in complication rates and neurological outcomes between the two study arms. Further studies should be conducted to define the positive and negative effects of this somehow novel technique in different populations as well.

## 1 Introduction

Acute spinal cord injury (SCI) resulting from traumatic events is one of the leading causes of disability in societies [1]. It mostly impacts the younger age population because of its most common cause [2] (which is vehicle collision.). Hence, it carries a tremendous socioeconomic and individual burden worldwide [[3], [4], [5]]. The injury consists of two types of injury: the primary and the secondary. Primary injuries result from the exact force and physical impact as a consequence of the trauma itself for example hemorrhage, axonal damage, vascular shearing, etc. Secondary injuries occur just after the primary damage and are a result of a cascade of signaling and downstream events which ultimately result in free radical formation, apoptosis induction, and inflammation. The recognition of secondary damage had led to various medical and surgical treatments [[6], [7], [8], [9], [10]].

Intravenous Corticosteroids, mainly methyl-prednisolone (MP) are probably the most debated and studied since the 60s. Several human clinical trials have been conducted throughout these decades for investigating the benefits and adverse systemic effects of MP in SCI the 3 staged NASCIS trail is the most famous. Despite these trials, and the benefits of MPSS acting as a neuroprotective agent, the role of MP in SCI is still controversial and experts suggest its use should be considered individually in each patient [[11], [12], [13], [14], [15]].

Outside this field of study, another form of corticosteroid has been suggested for the treatment of some conditions. Intrathecal administration of prednisolone has been utilized for postherpetic neuralgia and chronic complex regional pain syndrome [[16], [17], [18], [19], [20]]. Intrathecal administration of drugs is an important route for drug delivery. It bypasses the brain-blood barrier and acts exactly on the central nervous system, therefore, decreasing the total dose and reducing the systemic effects and further complications of the drug.

We aim to study the safety and efficacy of MP on acute SCI patients.

## 2 Patients and methods

This study was designed as a randomized, double-blinded, and prospective clinical trial. The study was single centered and took place at Imam Reza Hospital, Tabriz, Iran from 2014 to 2016. Acute traumatic patients diagnosed with acute SCI who met our inclusion and exclusion criteria enrolled in our trial. Then they would be grouped randomly into two interventional arms: Intrathecal and intravenous MP groups.

Inclusion criteria [1]: patients aged 18–75 years [2] acute traumatic etiology [3] thoracolumbar injury [4] written informed consent.

Exclusion criteria: Ref. [1] non-thoracolumbar cause of SCI [2] penetrating cord injuries [3] any condition which would not allow intervention in the first 8 h [4] previous spinal deformity [5] previously disabled patients [6] hemodynamically unstable [7] unconsciousness [8] any absolute or relative contraindication for Lumbar puncture [9] the use of high dose steroids within the last month before injury [10] serious medical condition which drug administration safety is unclear [11] patients with diabetes mellitus [12] Pregnant and breastfeeding patients [13] any systemic immunodeficiency or known infection [14] any relative or absolute contraindication for high-dose intravenous steroid [15] any condition which would interfere with consenting [16] patients with normal sensory and motor function.

Design: Each patient was assessed and treated based on our institutional protocol for spinal cord injury. Spinal and brain initial computed tomography (CT) was obtained for each patient based on the indication. Based on our institutional protocol, we obtained thoracolumbar MRI for every patient for further evaluation. Based on clinical examination, American Spinal Injury Association Impairment score (ASIA) for motor and sensory deficits and modified Frankel scores were measured and documented for each patient by at least two neurosurgery residents in the early hours of admission. The decision for surgery was based on institutional and individual senior attending decisions.

Intervention: two groups of intervention were designed as mentioned earlier: Intrathecal (IT) and intravenous (IV) arms. For the Intravenous group, based on previous studies, a bolus dose of 30 mg per kg MP was given intravenously in 15 min followed by 4–5 mg per kg which was infused based on the interval between injury and injection. Patients treated in the first 3 h after injury, were treated for 23 h, and patients treated after 3 h–8 h of injury were treated for 47 h.

On the IT treatment arm, patients we log rolled to lateral position and lumbar puncture was done ideally in the L4/5 interspinous space. After removing 1 mL of CSF, 1 mg per kg of MP was injected slowly, in the first 8 h after injury. This protocol was repeated 24 and 48 h later.

To better understand the biological bases of MP on SCI, we measured two biomarkers for oxidative stress in these patients as well. Serum malondialdehyde (MDA) and total antioxidant capacity (TAC) were measured in these patients at arrival and day 3 after injury.

Outcome: the efficacy of treatment was measured using changes in ASIA sensory and motor scores from baseline. Three checkpoints were chosen for assessment: baseline at admission, just before discharge, and 6 months after injury. Any adverse effects, complications, or death were evaluated throughout follow-up.

Randomization, blinding, and analysis: the statistical significance was chosen at p < 0.05, and the power was set to 80% (α = 0.05 and β = 0.2). Using similar previous studies [21,22] total sample size was estimated at 53 which for simplicity we concluded 60 patients overall. Using the R program, we used a blocked randomization technique (for equal treatment arm sizes) and a double blinding strategy was used. All analysis was done using SPSS ver 16. For comparison of within groups' results, we used repeated measure ANOVA, and for in-between measures, in each period we utilized paired *t*-test/Wilcoxon test.

## 3 Results

**Fig. 1:**
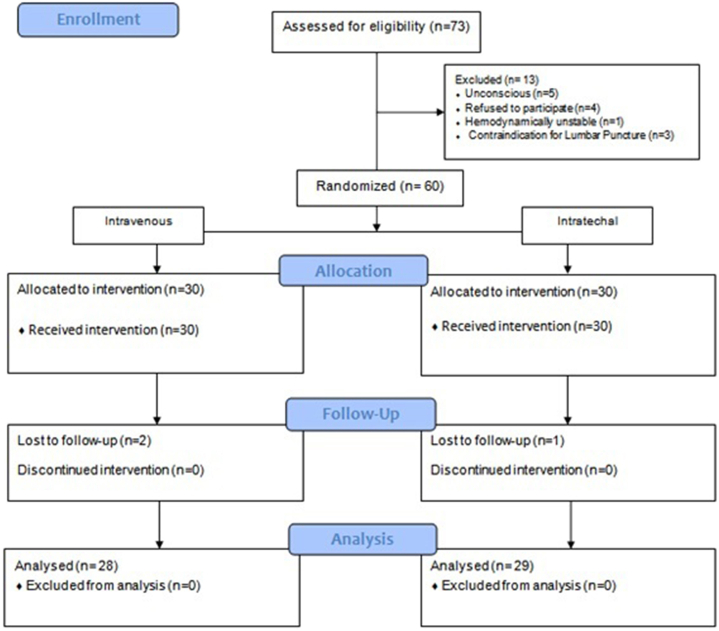
Flow diagram of patient allocation and analysis.

73 patients were diagnosed with acute SCI in Imam Reza hospital and 13 of them were excluded. The remaining 60 patients were randomized into two treatment arms accordingly. 3 patients (one from intrathecal and 2 from the intravenous group) were lost in follow-up. Therefore, ultimately the data of 57 patients were gathered and analyzed (Fig. 1).

### 3.1 Baseline data

The mean age of the IT and IV group was 26.10 ± 10.62 and 27.21 ± 11.05 respectively. Further analysis showed no significant difference between the ages (p = 0.79).

Overall, 8 patients were female (4 in each group) and 49 were male (25 for the IT group and 24 for IV) and there was no significant difference between the two groups statistically (p = 0.64).

### 3.2 Sensory evaluation

#### 3.2.1 Pinprick score

The total score for pinprick score at admission, on discharge, and after 66-month follow-up was 92.65 ± 10.87, 96.82 ± 10.7, and 100.03 ± 10.18 respectively (Table 1).

**Table 1:** Pinprick score in treatment groups over time.

	Admission	Discharge	Follow-up	P-value
Intravenous	93.43	97.14	99.96	0.000
Intrathecal	91.9	96.51	100.1	0.000
P-value	0.6	0.83	0.96	

**Fig. 2:**
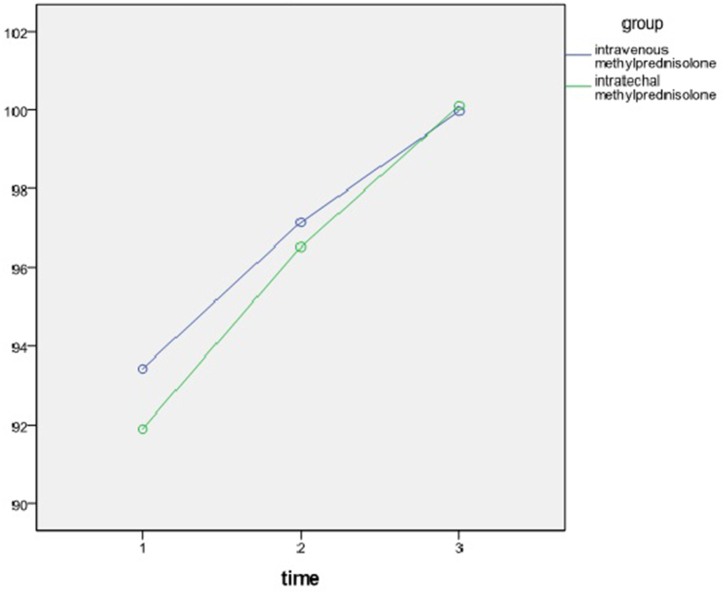
Pinprick scores over time.

A repeated measure ANOVA was conducted to compare the pinprick ASIA score of patients in two different treatment arms. There was a significant effect of time on pinprick score regardless of group (p < 0.000). The analysis showed that there was no interaction of time and group on light touch ASIA score and at every given time no significant difference was observed (p > 0.6) (Fig. 2).

#### 3.2.2 Light touch score

The total score for light touch at admission, on discharge, and after 6-month follow-up was 91.6 ± 11.4, 95.42 ± 11.13, and 99.67 ± 10.61 respectively (Table 2).

**Table 2:** Light touch scores in treatment groups over time.

	Admission	Discharge	Follow-up	P-value
Intravenous	92.5	95.75	98.78	0.000
Intrathecal	90.72	95.1	100.52	0.000
P-value	0.56	0.83	0.54	

**Fig. 3:**
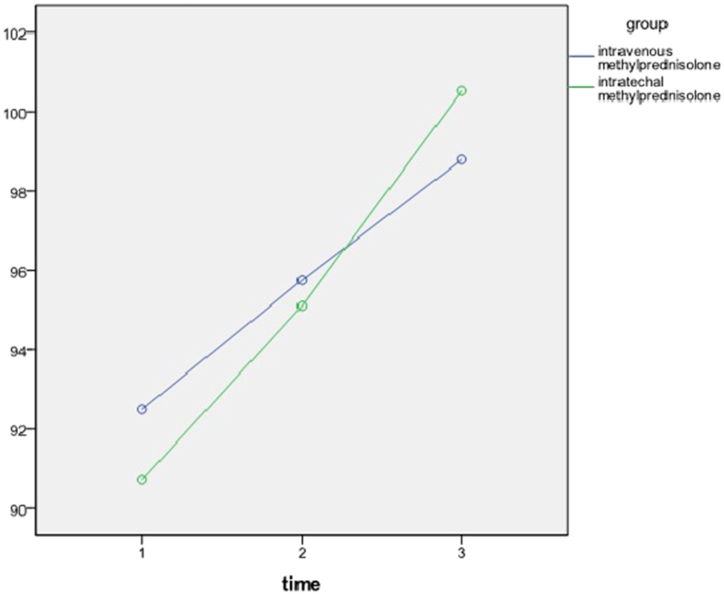
Light touch scores over time.

A repeated-measures ANOVA was conducted to compare the light touch ASIA score of patients in two different treatment arms. There was a significant effect of time on light touch ASIA score regardless of group (p < 0.000). The analysis showed that there was no interaction of time and group on light touch ASIA score and at every given time no significant difference was observed (p > 0.5) (Fig. 3).

### 3.3 Motor evaluation

The total Motor score at admission, on discharge, and after 6-month follow-up was 74.35 ± 11.58, 76.04 ± 12.16, and 77.75 ± 12.15 respectively (Table 3).

**Table 3:** Motor scores in treatment groups over time.

	Admission	Discharge	Follow-up	P-value
Intravenous	73.5	75.18	76.89	<0.005
Intrathecal	75.17	76.86	78.59	<0.005
P-value	0.59	0.6	0.6	

**Fig. 4:**
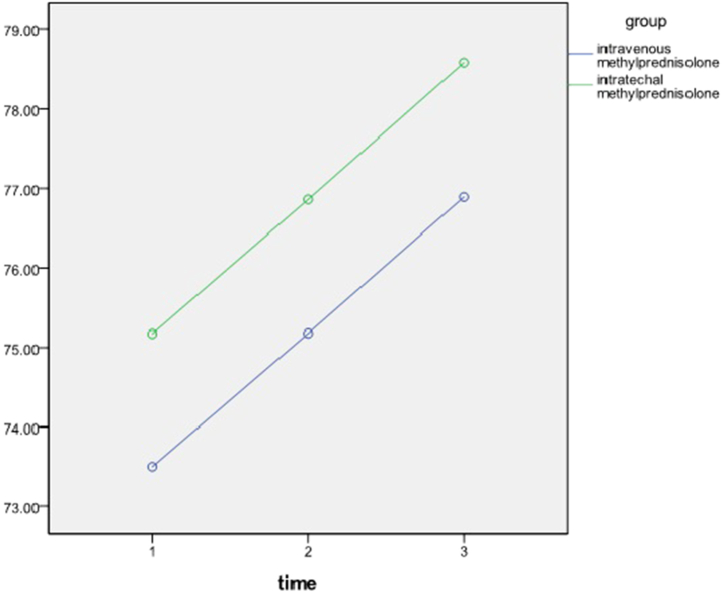
Motor scores over time.

A repeated-measures ANOVA was conducted to compare the ASIA motor score of patients in two different treatment arms. There was a significant effect of time on motor scores regardless of group (p < 0.005). The analysis showed that there was no interaction of time and group on light touch ASIA score and at every given time no significant difference was observed (p > 0.5) (Fig. 4).

### 3.4 Frankel score

Overall, at admission, 75% in the IV group and 65.5% in the IT were either Frankel A or B (Table 4).

**Table 4:** Frequency table of Frankel scores.

Frankel scores	Intravenous group			Intrathecal group		
	Admission	Discharge	6 m follow-up	Admission	Discharge	6 m follow-up
A	12 (42.9%)	8 (28.6%)	7 (25%)	12 (41.4%)	7 (24.1%)	7 (24.1%)
B	9 (32.1%)	7 (25%)	5 (17.9%)	8 (27.6%)	6 (20.7%)	6 (20.7%)
C	5 (17.9%)	8 (28.6%)	7 (25%)	7 (24.1%)	9 (31%)	7 (24.1%)
D	2 (7.1%)	4 (14.3%)	7 (25%)	2 (6.9%)	7 (24.1%)	7 (24.1%)
E	0 (0%)	1 (3.6%)	2 (7.1%)	0 (0%)	0 (0%)	2 (6.9%)

For within analysis of the Intravenous group, a Friedman's test showed a significant difference among Frankel scores in patients measured at admission, at discharge, and after a 6-month follow-up, X2F [2] = 26.755, p < 0.001. Post hoc test using a Wilcoxon signed-ranked test with a Bonferroni-adjusted alpha level of 0.017 (0.05/3) the analysis showed Frankel score improvement was significant at any given time (p < 0.017).

In the intrathecal group, within analysis showed a significant difference among Frankel scores in patients measured at admission, at discharge and after a 6-month follow-up, X2F [2] = 28.50, p < 0.001. Post hoc test using a Wilcoxon signed-ranked test with Bonferroni-adjusted alpha level of 0.017 (0.05/3) the analysis showed Frankel score improvement was significant between admission and any other time (P < 0.001) but not significant between discharge and 6 months follow up (p = 0.10).

Using multiple independent t-tests, a between-group analysis was done. The tests showed there were no significant differences among Frankel scores of two treatment arms in any given time (p values were measured at 0.78, 0.6, and 0.94 for admission, at discharge, and after 6 months of follow-up, respectively).

### 3.5 Serum MDA and TAC measurement

These two serum biomarkers were measured on admission and day three after injury. Data shows day zero MDA serum level of 2.12 ± 0.56 and 2.36 ± 0.52 for IV and IT groups respectively. Also, MDA serum levels on day 3 after injury were measured 2.08 ± 0.54 and 2.24 ± 0.64 for IV and IT groups respectively. Further analysis did not show any statistical difference between MDA levels in IV and IT groups in either admission (p = 0.1) or after three days (p = 0.32).

### 3.6 Complications

As shown in Table 5, five major complications were seen. The most common was urinary tract infection (UTI) which occurred in 15 patients (9 IV group and 5 IT group). Other complications included pneumonia, deep vein thrombosis (DVT), pulmonary embolism (PE), and gastrointestinal bleeding (GI bleeding). Further analysis showed no significant difference between these complications in the two groups (Table 5).

**Table 5:** Complications of each treatment arm.

	UTI	Pneumonia	DVT	PE	GIB
Intravenous	9	6	2	1	3
Intrathecal	6	2	4	2	1
P-value	0.33	0.11	0.41	0.55	0.28

## 4 Discussion

Intrathecal MP was first described in 1960 and was mainly used for treating Multiple sclerosis and sciatica pain [16]. Throughout the literature, the intrathecal administration of steroids-particularly MP- has been used for different diseases. Kotani et al. showed that there is a possible benefit for intrathecal injection of MP in postherpetic neuralgia (PHN) patients in a randomized trial [17]. But other studies following this study showed either no benefit or adverse effects and therefore this technique never became part of the standard treatment for PHN and remained somehow controversial [[17], [18], [19],^23^]. Chronic complex regional pain syndrome (CRPS) was another condition in which intrathecal MP was investigated. For instance, Munts et al. concluded that single bolus administration of intrathecal MP is not efficacious in chronic CRPS patients [20].

An important rationale for using the intrathecal route for MP in spinal cord injury is that studies have shown systematic injections do not lead to a measurable amount of MP in CSF [24,25]. The reason hypothesized for this is the effect of the brain-blood barrier (BBB), particularly a protein called P-Glycoprotein. This protein is an efflux transporter that acts on MP as a substrate and reduces the bioavailability of MP in CSF [23,24]. Our present understanding of MP in CSF is complex and incomplete. Studies suggest that MP is hydrolyzed by cholinesterase once injected intrathecally [23,26]. Free MP has three main routes: entering cells, reaching the systemic circulation, and getting metabolized [23]. Both animal and human studies have investigated the peak plasma concentration of MP after intrathecal injections. These studies showed a peak in plasma concentration after 24 h and measurable amounts after 21 days following 80 mg intrathecal injection (8,130). This could be the possible explanation for systemic effects and complications of intrathecal treatments.

The present study was designed to evaluate the effect of using intrathecal compared to intravenous steroids in acute spinal cord injuries. Accordingly, we observed favorable results in both sensory and motor scores after 6 months in each treatment arm. Our results failed to demonstrate any superiority of the two treatment arms over each other. Moreover, our results did not suggest any difference in complication rates among the groups. We did not include patients with prior spinal deformity due to potential side effects of lumbar puncture. The existence of concomitant traumatic brain injury (TBI), which may result in elevated intracranial pressure, is another significant clinical complication that should be taken into account in trauma patients (ICP). Patients with TBI were not included in this study if imaging or clinical examinations revealed any indications of elevated ICP.

This study has several limitations. First, surgical treatment strategies and indications for surgery were not standardized and despite being a single-center study, it could vastly influence our conclusion. Second, we did not have a control group to compare the baseline effect of MP. Due to the previous controversy in the effectiveness of steroids, utilizing a control group could result in a better interpretation of the results. Third, our population was relatively young and as we know from previous studies, the recovery rate is higher in younger patients [[27], [28], [29], [30]]. Due to our sample size, we could not analyze older patients separately without affecting the validity of our results.

## 5 Conclusion

This is the first known clinical trial investigating the effect of intrathecal MP in acute SCI patients. Our finding did not show any significant differences in complication rates and neurological outcomes between the two study arms. Further studies should be conducted to define the positive and negative effects of this somehow novel technique in different populations as well.

## Ethics

The study was approved by the Research Ethics Committee of Tabriz University of Medical Sciences (IR.TBZMED.REC.1396.971).

## Author contribution statement

Ali Meshkini: Conceived and designed the experiments; Contributed reagents, materials, analysis tools or data; Wrote the paper.

Mohammad Kazem Sarpolaki: Analyzed and interpreted the data; Wrote the paper.

Ali Vafaei: Contributed reagents, materials, analysis tools or data; Wrote the paper.

Farhad Mirzaei: Conceived and designed the experiments; Performed the experiments.

Abolfazl Badripour; Ebrahim Rafiei; Mohammad Reza Fattahi: Performed the experiments; Contributed reagents, materials, analysis tools or data.

Morteza Khalilzadeh: Conceived and designed the experiments; Performed the experiments; Contributed reagents, materials, analysis tools or data.

Arad Iranmehr: Conceived and designed the experiments; Analyzed and interpreted the data; Contributed reagents, materials, analysis tools or data; Wrote the paper.

## Data availability statement

Data will be made available on request.

## Clinical trial registration

Iranian Registry of Clinical Trials approval was obtained before initiating the study (IRCT number: IRCT20190116042374N1).
